# The Effect of Yellow and White Lupine Meals on the Growth Performance, Carcass Composition, and Meat Quality of Fleckvieh Finishing Bulls

**DOI:** 10.3390/ani15060790

**Published:** 2025-03-11

**Authors:** Luděk Bartoň, Daniel Bureš, Nicole Lebedová, Filip Jančík, Magdaléna Štolcová, Jerico Consolacion, Tersia Kokošková

**Affiliations:** 1 Department of Cattle Breeding, Institute of Animal Science, Přátelství 815, 104 00 Prague, Czech Republic or buresd@af.czu.cz (D.B.); lebedova.nicole@vuzv.cz (N.L.); stolcova.magdalena@vuzv.cz (M.Š.); 2 Department of Food Quality, Faculty of Agrobiology, Food and Natural Resources, Czech University of Life Sciences Prague, Kamýcká 129, 165 00 Prague, Czech Republic; 3 Department of Animal Feeding and Nutrition, Institute of Animal Science, Přátelství 815, 104 00 Prague, Czech Republic; jancik.filip@vuzv.cz; 4 Department of Animal Science and Food Processing, Faculty of Tropical AgriSciences, Czech University of Life Sciences Prague, Kamýcká 129, 165 00 Prague, Czech Republic or jerico.consolacion@msunaawan.edu.ph (J.C.); kokoskova@ftz.czu.cz (T.K.); 5 Department of Agricultural Sciences, College of Environment and Life Sciences, Mindanao State University at Naawan, Naawan 9028, Philippines

**Keywords:** blood biochemistry, carcass traits, cattle, fatty acid, feed intake, lupine seed, sensory analysis

## Abstract

The aim of the study was to investigate the incorporation of white (WL) and yellow lupine (YL) seed meal as a replacement for rapeseed meal (RS) in the diets of finishing bulls with respect to growth, serum biochemical parameters, carcass composition and meat quality, including sensory analysis. A total of 30 Fleckvieh bulls (average initial weight 441 kg; SD = 33 kg) were housed under identical conditions and fed ad libitum diets similar in protein and energy contents, but with different protein sources—YL seed meal (70 g/kg DM), WL seed meal (77 g/kg DM), and RS meal (75 g/kg DM)—for an average of 85 days. The RS bulls gained weight more rapidly (*p* < 0.05) and were more efficient (lower feed to gain ratio; *p* < 0.001) than the YL animals, with the WL group being intermediary. The slaughter and carcass traits, most blood parameters, and meat quality attributes measured in two different muscles were not affected by diet. The proportions and contents of saturated and monounsaturated fatty acids were higher in YL and WL meat (*p* < 0.001), whilst polyunsaturated fatty acid proportions were higher in RS meat (*p* < 0.001). Several meat texture characteristics were slightly more favourable in the YL samples compared to the other treatment groups. In conclusion, WL could be used as a replacement of RS in the diets for finishing bulls. However, further research is needed to examine different varieties and inclusion levels of YL in cattle feeding.

## Simple Summary

Grain legumes have the potential to become a sustainable alternative to other protein sources like soybean and rapeseed. Important representatives of the grain legumes used as livestock feed are certain species of lupines, which are characterised in terms of nutrient composition by their high protein and oil content. An experiment was conducted to examine the use of yellow and white lupine seed meals as a substitute for rapeseed in the diet of finishing bulls, and their effects on growth, carcass composition, and beef quality. Animals fed yellow lupine meal had lower live weight gains and inferior feed efficiency compared to the control rapeseed-fed group, while the results for white lupine were intermediary. All groups achieved comparable results for carcass composition and most meat quality traits. The proportions of fatty acids that are considered beneficial in terms of potential cardiovascular disease risk to consumers were lower in the meat of lupine-fed bulls.

## 1. Introduction

European legume production is currently marginal, with grain legumes covering less than 3% of arable land [1]. However, as indicated in the EU Common Agricultural Policy Strategic Plans (2023–2027), support for protein crops and legumes will increase by 25% compared to 2022. The supported area is, thus, estimated to reach 7 million hectares in 2027 [2]. Grain legumes have the potential to contribute more to European agricultural systems by improving the agronomic performance of cropping systems, providing protein-rich food and feed, and helping to reduce the European dependence on imported protein [3]. Furthermore, using grain legumes in intensive crop rotation systems with cereals may reduce environmental burdens due to the decreased application of inorganic nitrogen fertilisers, reduced tillage, and greater diversification of the crops in rotation [4]. In spite of the clear benefits for environmentally friendly production systems and protein supply, economic constraints must be overcome to support the transition processes for legume reintegration [1].

Worldwide, there are a large number of lupine species, but those used as livestock feed include predominantly white lupine (*Lupinus albus*), narrow-leafed lupine (*Lupinus angustifolius*), and yellow lupine (*Lupinus luteus*) [5]. Of these three species, higher seed yields per ha were observed for white lupine than for yellow and narrow-leafed lupine species [6,7]. Compared to cereals and most other legume grains, lupine grains contain a high protein content (>300 g/kg) and low starch content (<20 g/kg). They are also high in oil (60–100 g/kg) rich in the essential fatty acids C18:2 n-6 and C18:3 n-3 [7,8].

The use of lupine species has been limited in the past by the high content of alkaloids, which cause a bitter taste. However, as a result of breeding efforts, current cultivars have lower alkaloid levels. Current lupine breeding is targeted at yield stabilization, resistance to biotic and abiotic stresses, and the improvement of seed quality characteristics, such as the content of non-starch poly- and oligosaccharides [9].

While the use of lupines in dairy cow diets has been extensively investigated and reviewed [8,10], limited information is available on its use for growing and fattening beef cattle. It has been previously reported that white lupine meal can effectively substitute soybean meal in the diets of Charolais heifers [11] and young Podolian bulls [12]. To the best of our knowledge, the effect of feeding yellow lupine grain on cattle growth and beef quality has not been examined. Rapeseed is currently one of the most popular oil crops in Europe, with multiple applications as human food and animal feed and for industrial purposes [13]. Rapeseed meal, a by-product of crude rapeseed oil extraction, contains high levels of digestible protein (25–40%) [14]. As a result, it can be effectively used to replace soybean meal in the diets for feedlot cattle without negative consequences on growth performance and carcass characteristics [15]. Therefore, this study was conducted to evaluate the effects of replacing rapeseed meal with white and yellow lupine seed meals in the finishing diets of Fleckvieh bulls on their growth performance, blood biochemical parameters, carcass composition, and beef quality.

## 2. Materials and Methods

### 2.1. Animals and Diets

Experimental procedures were approved by the Animal Care Committee of the Ministry of Agriculture of the Czech Republic (No. MZE-58151/2022-13143). A total of 30 Fleckvieh bulls, the progeny of 20 different sires, were used in the experiment. They were purchased from a commercial herd and transported at an average age of 232 (SD = 8.0) days to the experimental stable of the Institute of Animal Science in Prague (IAS), Czech Republic. They were loose-housed in three identical straw-bedded pens and trained to feed from electronically controlled feeding stations (Hokofarm Group, Marknesse, the Netherlands). During training, all animals had ad libitum access to an identical mixed diet based on maize silage, alfalfa silage, concentrate mixture, and mineral–vitamin mixture with a concentrate/forage ratio of 45/55. The diet was delivered to the feeding stations four times a day.

At an average age of 336 (SD = 8.3) days and an average live weight (LW) of 413 (SD = 31.8) kg, the bulls were divided into three dietary treatment groups of 10 animals each, balanced for LW and age. For an adaptation period of 22 days, and then for the entire trial period until slaughter, they received ad libitum diets similar in protein and energy contents but differing in protein sources, either yellow lupine seed meal (YL; *Lupinus luteus cv Salut*), white lupine seed meal (WL; *Lupinus albus cv Amiga*), or extruded rapeseed (RS; *Brassica napus*) meal. The RS meal in the control diet (75 g/kg DM) was replaced by YL meal (70 g/kg DM) and WL meal (77 g/kg DM) in the experimental diets. The seeds of both lupine varieties were treated by crushing in a hammer crusher with a 5 mm mesh screen. The composition of the three total mixed diets and the YL, WL, and RS meals is given in Table 1. Diet ingredients were dried at 55 °C for 48 h and residual moisture was determined by oven drying for 6 h at 105 °C. Ash was determined after 6 h at 550 °C and ether extract after a 6 h extraction process with petroleum–ether using Soxtec 1043 (FOSS Tecator AB, Höganäs, Sweden). Nitrogen was determined using the Kjeldahl method (Kjeltec AUTO 1030 Analyser, FOSS Tecator AB, Höganäs, Sweden) [16], and crude protein was calculated as N × 6.25. The acid detergent fibre content was determined in accordance with AOAC International [16]. The neutral detergent fibre content was analysed in the presence of sodium sulphite and with α-amylase treatment [17] and is presented as ash-free. Fibre fractions were determined using Fibertec 2010 (FOSS Tecator AB, Höganäs, Sweden).

### 2.2. Animal Performance, Slaughter, and Carcass Characteristics

After a diet adaptation period of 22 days, the trial period started, and it lasted for an average of 85 days until slaughter. The animals were weighed at the same time of day (08:00 a.m.) without prior fasting at the beginning (initial weight) and end (slaughter weight) of the trial period, and every two weeks during the trial. Individual feed intakes were measured daily to determine DM intake (kg DM/day) and feed-to-gain ratio (F:G; DM intake/daily gain). One bull from the WL group had to be excluded from the experiment due to severe laminitis.

The bulls were slaughtered at the IAS experimental slaughterhouse located 2 km from the stable. The slaughtering started after 63 days of the trial. The two heaviest animals from each group were selected for slaughter on each of the five slaughter days (one slaughter day per week due to the slaughterhouse capacity), except for slaughter day 3 when only one bull from the WL group was slaughtered. The animals were stunned with a captive bolt pistol and killed by exsanguination. Within 1 h after slaughter, the carcasses were uniformly dressed, split, and assessed by a trained classifier for conformation (on an 18-point scale) and fatness (15-point scale) class according to the EU beef carcass classification scheme with the use of subclasses [20]. Total internal fat weights (sum of kidney, stomach, heart, and cod fat) were recorded. Dressing percentage was calculated from the difference between the slaughter weight and hot carcass weight. After cooling for 48 h at +2 °C, the right carcass halves were divided into standardised joints and their weights were recorded. In accordance with the prevailing European practice, the loin was separated between the 5th and 6th ribs [21] and further dissected into meat, trimmings, bone and tendons, and separable fat. Only the cleaned section of *longissimus thoracis* (LT; between the 6th and 13th ribs) and the whole cleaned *longissimus lumborum* muscles were considered as meat; the remaining muscles were evaluated as trimmings. Between the fifth and sixth ribs, a picture was obtained for each loin sample, with a ruler next to it to calibrate the image, and then, the LT muscle area was measured using the image analysis software NIS Elements AR 3.2. (Nikon Instruments Europe B.V., Amsterdam, the Netherlands).

### 2.3. Blood Sample Collection and Analyses

Two blood samples were collected from each bull at the beginning (Sampling 1) and on day 63 of the trial (Sampling 2) from the coccygeal vein into 4 mL BD Vacutainer^®^ rapid serum tubes (Becton Dickinson, Franklin Lakes, NJ, USA) at the same time on each sampling day (7–8 a.m.). After collection, the blood was allowed to coagulate by leaving it undisturbed at room temperature [22]. Thereafter the serum was transferred into clean 1.5 mL Eppendorf^®^ Safe Lock microtubes (Eppendorf, Hamburg, Germany) using a Pasteur pipette. Serum samples were stored at −18 °C until biochemical analysis, which was performed at the Large Animal Clinical Laboratory of University of Veterinary Science Brno, Czech Republic. The sera were analysed for concentrations of total protein (TP), albumin (ALB), triacylglycerol (TG), cholesterol (CHOL), urea and creatinine (CREAT), activity of aspartate aminotransferase (AST), gamma-glutamyl transferase (GGT), and alkaline phosphatase (ALP). All analyses were carried out by photometric method in an automatic biochemical analyser (Konelab 20XT, ThermoFisher Scientific, Waltham, MA, USA) using commercial BioVendor (BioVendor—Laboratorní medicína a.s., Brno, Czech Republic) and DiaSys kits (Diagnostic Systems GmbH, Holzheim, Germany). The total globulin (GLB) fraction was determined by subtracting ALB from TP and then, the ALB to GLB ratio (AGR) was calculated [23].

### 2.4. Meat Sample Collection and Analyses

At 48 h after slaughter, the LT and *rectus abdominis* (RA) muscles were removed from the chilled right carcass halves and transported in a cooler box to the lab for further processing. The section of the LT muscle between the 6th and 9th thoracic vertebrae was used for physical and chemical measurements, and the section between the 9th and 11th thoracic vertebrae was used for sensory analysis. The RA muscle was collected in its entirety; the central part was used for sensory analysis and physical properties measurements, and the peripheral parts were used for chemical analysis.

The meat pH values were obtained using a spear probe (CHS Cultro Dart) attached to a Vio DSH FOOD pH7 pH meter (Giorgio Bormac s.r.l., Capri, Italy) 48 h after slaughter. Instrumental colour was measured with a portable spectrophotometer (CM-2500d, Konica Minolta, Osaka, Japan) using CIE scores (Comission Internationale de L’eclairage). The measurements were taken in triplicate at three different locations on the muscle cut after 30 min of air exposure. Colour data (L*, lightness; a*, redness and b* yellowness) were presented as averages of these three locations for statistical analysis. Colorimeter parameters included a circular measurement area of 8 mm diameter; specular component 0% UV, standard illuminant D65, which simulated daylight; 10° observer angle, and zero and white calibration.

Shear force was measured on an Instron Universal Texture Analyzer 3365 (Canton, MA, USA) using a Warner-Bratzler blade. The samples were cut into 20 mm thick slices and heat-treated according to the method of Honikel [24]. After heat-treatment and cooling down to room temperature, they were cut into 10 × 10 × 20 mm prisms, which were sliced across the muscle fibres at a 90° angle and a crosshead speed of 100 mm/min. The value for one sample was calculated as the average of six cuts. Additionally, meat cooking loss was determined as the sample weight difference before and after heat treatment.

The analysis of meat chemical composition was performed in accordance with the procedures of AOAC International [18]. The samples intended for chemical composition analyses were homogenised in a food blender and frozen at −20 °C until analyses. Dry matter content was determined by oven drying at 105 °C to a constant weight and comparing the initial and final weights. A Grindomix GM200 knife mill (Retsch, Haan, Germany) was used to pulverize the dried samples and crude protein was analysed using a Kjeltec 2400 (FOSS Tecator AB, Höganäs, Sweden). The Soxhlet method described in ISO 1444 [25] was used to analyse intramuscular fat, through extraction with hexane (Soxtec Avanti 2055, FOSS Tecator AB, Höganäs, Sweden), while crude ash content was determined by incinerating samples at 550 °C using an electric furnace (LAC L15/12, LAC, Židlochovice, Czech Republic) for 24 h.

Fat-free dry matter was used to determine the collagen content as described in our previous study [26]. Soluble (heat-labile) and insoluble collagen were determined by measuring the hydroxyproline content of duplicate 1 g samples. Collagen solubility was calculated as the percentage of soluble collagen as a fraction of total collagen.

The fatty acid (FA) proportions (g/100 g FA determined) and contents (mg/100 g muscle) of the LT samples were determined by gas chromatography after the extraction of the total lipids, as described in our previous study [27]. The index of atherogenicity (IA) and index of thrombogenicity (IT) were calculated according to Ulbricht et Southgate [28].

The LT and RA muscle samples intended for sensory analysis were vacuum-packed in a plastic bag and aged at +4 °C until 14 days after slaughter. Then, they were frozen and stored at −20 °C for approximately three months until sensory analysis. One day before each sensory evaluation, the samples were allowed to thaw at laboratory temperature inside the plastic packaging. Then, 20 mm thick slices of LT samples and the central part of the RA muscle, weighing approximately 400 g, were grilled on a double-sided glass–ceramic grill (VCR 6l TL, Fiamma, Aveiro, Portugal) tempered to 200 °C, to a final internal temperature of 70 °C. Subsequently, the samples were cut into approximately 20 × 20 × 20 mm cubes, which were placed in glass containers and kept at +50 °C for approximately 1 h until evaluation. Samples were coded using a three-digit random code. The sensory analysis itself consisted of four sessions during which a total of 58 samples were provided to the assessors trained according to ISO 8586 [29]. In one session, a maximum of 15 samples were presented simultaneously to 10 trained panellists in five separate sets in random order. Each set consisted of three samples of the same muscle from three animals differing in dietary treatment with the exception of one set that consisted of only two samples. The evaluation was carried out in a sensory laboratory equipped with 10 individual booths. Colour discrimination of the samples was prevented by the application of red lighting. A total of 11 descriptors were assessed (Table 2) using a 100 mm long unstructured scale, which was converted for statistical purposes to a numerical scale (0 to 100). Bread and water were available to the assessors as palate cleansers.

### 2.5. Statistical Analyses

Statistical analyses were performed using the SAS package (ver. 9.4; SAS Institute Inc., Cary, NC, USA). All variables were tested for normality using Kolmogorov–Smirnov goodness of fit test and for homogeneity of variance using Levene’s test. A general linear model (procedure GLM) with the fixed effect of dietary treatment group (experimental unit, n = 3), and the initial weight at the beginning of the trial (overall mean 441 kg) as a covariate, was used for analysing the growth and feed intake traits of 29 bulls (observational units). Blood parameters were analysed using a GLM with the fixed effect of dietary treatment separately for the data from each of the two sampling days. For evaluating carcass, meat quality and sensory traits, a linear mixed model (procedure MIXED) was fitted using REML (restricted maximum likelihood) with the fixed effect of dietary treatment. The model for carcass and meat quality traits included the day of slaughter as a random effect, while day of slaughter, sensory session, and assessor were included as random effects in the model for sensory characteristics. The Kruskal–Wallis test was used for not normally distributed data (conformation, fatness, AST and CK in blood Sampling 1, and CREAT, UREA, CK, CHOL, and TG in blood Sampling 2). Significance of differences between groups was tested by Tukey’s post hoc test and by Dwass, Steel, Critchlow–Fligner multiple comparison procedure for not normally distributed data. Data are presented in tables as least squares of the mean (LSM) with standard error (SEM; n = 9). Significance was declared if *p* < 0.05 and tendencies were considered at 0.05 ≤ *p* < 0.10.

## 3. Results

### 3.1. Animal Performance

Growth performance, feed intake, and feed efficiency data are presented in Table 3. The RS bulls had a higher slaughter weight than both WL and YL bulls, with WL bulls being heavier than YL animals (*p* < 0.001). The RS bulls gained weight more rapidly (*p* < 0.01) and more efficiently (lower F:G; *p* < 0.001) than the YL animals. No differences were found in daily DM intakes.

### 3.2. Blood Biochemical Parameters

Blood biochemistry data are reported in Table 4. At the beginning of the test period (Sampling 1), there were no significant differences between treatments. The control RS group tended to have reduced levels of GGT (*p* = 0.073) and TG (*p* = 0.069). The concentration of urea was higher in the WL compared to the YL group (*p* < 0.05) at day 63 of the test period (Sampling 2). In addition, tendencies towards the highest levels of albumin in the WL (0.066) and AST in the YL group (*p* = 0.057) were observed.

### 3.3. Slaughter and Carcass Characteristics

The effects of different dietary treatments on slaughter and carcass characteristics are presented in Table 5. Hot carcass weights were lowest in the YL group and highest in the RS group (*p* < 0.001). The RS bulls received higher conformation classification scores than the YL bulls (*p* < 0.05). No significant differences among the groups were observed in dressing percentage, internal fat, and loin composition. In addition, the dietary groups did not differ in the weight of different carcass joints nor when they were considered as a proportion of the hot carcass weights.

### 3.4. Physical Meat Quality Parameters and Chemical Composition of Muscle Samples

The physical meat quality and chemical composition parameters of LT and RA muscles are shown in Table 6. A significant result was detected for the LT pH values measured 48 h after slaughter (*p* = 0.049) but the Tukey post hoc test failed to identify any differences between treatment means. No significant differences between dietary treatments were found in any of the remaining traits listed in Table 6. It should be noted, however, that although insignificant, intramuscular fat content was higher in both muscles from the WL bulls compared to the other groups.

### 3.5. Fatty Acid Composition of Muscle Samples

The FA proportions and contents measured in the LT are shown in Table 7. Only major and nutritionally important FAs representing more than 92% of the total FAs are presented. Higher proportions of C14:0, C18:0, total saturated fatty acids (SFA), C14:1 n-5, C18:1 n-9, total monounsaturated fatty acids (MUFA), and the MUFA/SFA ratio (*p* < 0.05) were observed in the meat from lupine-supplemented YL and WL bulls compared to the RS group. Individual and total polyunsaturated fatty acid proportions (PUFA), as well as the PUFA/SFA ratio, were higher (*p* < 0.05) for the control RS group. Both nutritional indices (IA and IT) were lower in RS compared to YL and WL meat (*p* < 0.001). The contents of individual and total SFA, C14:1 n-5, C18 n-9, total MUFA, and total FA were higher in WL meat than in RS meat (*p* < 0.05), with YL being intermediary. No significant differences among the dietary treatment groups were found for PUFA.

### 3.6. Sensory Properties of Meat

Dietary treatment effects on the sensory properties of grilled LT and RA samples are presented in Table 8. Compared to RS bulls, the LT samples from WL bulls scored higher for juiciness (*p* < 0.05). As for RA samples, the YL group received higher scores for tenderness (*p* < 0.01) and nutty flavour (*p* < 0.05) compared to the WL group, and for chewability (*p* < 0.05) compared to the RS group.

## 4. Discussion

The Fleckvieh (Simmental) breed used in the present experiment is a late-maturing dual-purpose breed with economically sustainable, high-quality milk and meat production, and can be successfully intensively fattened to high final live weights [30]. Because of these characteristics, the breed has spread from Central Europe to almost all over the world. As described in our earlier study, when compared with other breeds, the carcasses of fattened Fleckvieh bulls are characterised by good conformation and relatively low carcass and intramuscular fat deposition [31].

While the effect of feeding white lupine grain meal on cattle growth performance has been examined in several previous studies, we were unable to find any reports focusing on yellow lupine. In the present study, the growth performance parameters of the YL group were lower compared to those of the RS group. The substantial magnitude of these differences was not expected, and it is difficult to explain, warranting further investigation. The current results are, to a certain extent, in agreement with the results of Wiese et al. [32] who reported lower, yet insignificant, live weight gains in lambs fed narrow-leafed lupine grain compared to those given RS meal. No differences in production performance were found by Vicenti et al. [12] and Ragni et al. [11], who examined the effect of white lupine and soybean meal supplementation in the diets of growing cattle. In addition, feeding narrow-leafed lupine seeds compared to RS meal reduced slaughter weight and average daily gain in Simmental bulls, apparently due to a higher feed intake in the RS-fed group [33]. However, in the present study, feed intake was similar in all groups. Similarly, beef steers fed soybean meal had higher daily gains than those given raw lupine in the growing, but not the finishing, phase [34]. In contrast, no significant differences in growth performance were reported when low amounts of yellow and white lupines were included in lamb diets [35]. It has been previously reported that raw lupine seed protein is highly soluble and degradable in the rumen [36]. Therefore, the reason for the lower growth performance of especially young animals fed lupine-containing diets could be the reduced intestinal availability of undegraded rumen protein. In the present study, however, older animals with live weights exceeding 400 kg were used. The nutritional value of raw feed components can be effectively increased by treatments such as extrusion, toasting, or extraction [37]. The lack of heat treatment of YL and WL seeds may have also contributed to the reduced growth performance of YL and WL animals. The quality of lupine proteins is also decreased by the deficiency of several essential amino acids, especially sulphur-containing methionine [38], which may have also affected the weight gains of bulls in this study.

Although bitter-tasting alkaloid concentrations in lupine seeds were not measured in this study, they apparently did not limit feed intake, which was similar in all groups. Indeed, the white lupine variety Amiga used in the present experiment has been previously characterised as sweet with an alkaloid content less than 0.5 g/kg dry matter [39].

At the beginning of the experiment (Sampling 1), no significant differences in blood biochemical parameters were detected between the groups, indicating that the groups were well-balanced physiologically. At day 63 of the test period (Sampling 2), there was a notable effect, especially on the protein profile, with a lower urea content observed in the YL group compared to the WL group. However, the measured blood urea concentrations were within the reference range (0.33 to 4.50 mmol/L in cattle; [40]), except for Sampling 2 in the WL group, who had a slightly higher average urea concentration of 4.76 mmol/L. Reduced serum urea concentration and a tendency towards lower albumin concentrations in the YL group might be explained by the lower degradability of organic matter in yellow lupine compared to white lupine meals, as reported by Musco et al. [5].

The RS group was scored higher for carcass conformation compared to the YL group, probably as a result of significantly higher hot carcass weights observed for the RS animals. Indeed, a strong positive relationship between carcass conformation and carcass weight has been previously reported [41]. In agreement with the present study, no effect of lupine seed diets on cattle slaughter and carcass data has been observed in earlier works [11,12].

Muscle pH is one of the most important indicators of meat quality. The average pH values observed in this study fell within the range for normal ultimate pH measured in beef muscle (5.5–5.8). None of the samples had a pH value higher than 6.2, which is the threshold indicating dark cutting beef [42]. Slightly higher pH values observed for the RA muscle may be related to the specific muscle fibre composition (lower amount of fast glycolytic fibres) [26]. No significant differences between dietary groups were detected for any of the physical and chemical parameters measured in the LT and RA muscle in the present study. Other studies using lupines in cattle diets have also found no effect on meat physical and chemical composition traits [11,12,43]. These results further support the statement that protein supplementation in the diet of fattening bulls has a limited effect on meat quality traits [44]. Similarly, no differences were observed for meat quality attributes when soybean meal was replaced with white and yellow lupine seeds in lamb diets [45].

The FA composition of lupine seeds and rapeseed meal used in this experiment corresponded to that reported in other studies [5,33], with the predominant FA being C18:2 n-6 in YL and C18:1 n-9 in WL and RS. However, the differences in diet FA composition were only partially reflected in the FA composition of muscle lipid fraction due to the extensive lipolysis and subsequent biohydrogenation of unsaturated FA occurring by the action of ruminal bacteria [46]. The current results are in agreement with those of Sami et al. [33], who observed increased total PUFA and C18:3 n–3 concentrations in beef from bulls fed rapeseed meal compared to those given lupine seeds. In other studies comparing cattle diets supplemented with either white lupine or soybean, no differences in FA composition of intramuscular fat were found, with the exception of higher C18:2 n-6 obtained for the soybean group [11,12]. The present study, on the other hand, revealed significant differences in meat fatty acid composition, particularly between the control and both lupine-fed groups. Whereas the proportions of SFA and MUFA were higher in YL and WL meat, both n-6 PUFA and n-3 PUFA proportions were higher in RS meat. This may be partially explained by a lower total FA content in the RS group compared to the other groups. It has been previously reported that the content of SFA and MUFA increases faster with increasing total FA content than the content of PUFA does. Therefore, the relative proportion of PUFA and the PUFA/SFA ratio decrease with increasing total FA content [47]. Nutritional indices IA and IT were used to assess the potential effects of FA composition on the cardiovascular health of consumers. The consumption of meat with lower IA and IT, as observed in the RS group, may be associated with a reduction in the risk of coronary heart disease development, but no recommended values for IA and IT have been determined yet [48].

The LT and RA muscles differ in size, metabolic activity, and culinary use, and were, thus, used to evaluate the effect of the experimental diets on meat sensory properties. When the organoleptic characteristics of the LT and RA muscles in Fleckvieh bulls were compared, it was found that the LT muscles showed more favourable scores in texture characteristics, i.e., fibrosity and chewiness [26]. To the best of our knowledge, the effect of lupine seed meal supplementation on the sensory parameters of beef has not yet been published. Ragni et al. [49] investigated the sensory quality of loins from the lambs fed lupine meal, and compared to animals fed soybean meal, there was a higher overall odour intensity, but no difference in meat texture parameters. On the other hand, Volek et al. [50] examined the sensory characteristics of the *longissimus lumborum* muscle from rabbits supplemented with either soybean meal or dehulled white lupine meal, and the lupine-fed group showed significantly higher values for meat tenderness and fibrosity. This is partly in agreement with the current study, where more favourable textural characteristics of meat were found, especially in the YL group. Positive effects of feeding field pea, another grain legume, on meat textural characteristics have been reported [51,52,53] but the results were not conclusive. The improvement in tenderness associated with field pea feeding was explained by a reduction in calpastatin production in muscle tissue, which led to an increased efficiency of calpain in the process of *postmortem* fragmentation of muscle fibre protein chains. However, further research is needed to clarify the possible positive impact of feeding grain legumes on the textural characteristics of beef.

## 5. Conclusions

The present study provides data on the effect of replacing RS meal with WL and YL seed meal in finishing bull diets on their growth performance, blood parameters, carcass composition, physicochemical quality, and the sensory properties of beef. The limitations of the study were the relatively small sample size and the need to slaughter animals on multiple slaughter days. In summary, reduced live weight gain, lower efficiency measured by F:G, hot carcass weight, and carcass conformation were observed especially in YL-fed compared to RS-fed animals, with WL-fed bulls being intermediary, whereas few differences among groups were seen for physical parameters and the chemical composition of meat. The two lupine groups were clearly distinguished from the control group in muscle fatty acid composition, especially in terms of increased MUFA and SFA and lower PUFA proportions. A certain tendency towards improved meat textural characteristics was found in the YL group. While YL and WL have been confirmed as valid alternatives to RS in diets for finishing bulls in terms of most carcass composition and meat quality parameters, they failed to replace RS in terms of growth performance and feed efficiency. The possibility of using YL has been explored for the first time and further research will be needed to test other varieties of YL and how they can be incorporated into diets for finishing cattle.

## Figures and Tables

**Table 1:** Ingredient and nutrient composition of the experimental diets.

	Treatment Group		
Item	YL ^1^	WL ^2^	RS ^3^
Diet ingredient composition (% DM)			
Maize silage	50.5	50.1	50.2
Alfalfa silage	8.7	8.7	8.7
Wheat straw	3.4	3.4	3.4
Wheat grain meal	26.3	26.1	26.2
Oat grain meal	2.6	2.56	2.6
Yellow lupine grain meal	7.0		
White lupine grain meal		7.7	
Rapeseed meal			7.5
Vitamin–mineral supplement with urea ^4^	1.4	1.4	1.4
Diet nutrient composition			
Dry matter (% fresh weight)	52.3	52.5	52.3
Crude protein (% DM)	13.3	13.2	13.2
Organic matter (% DM)	94.7	94.8	94.3
Ether extract (% DM)	3.5	4.0	3.0
Neutral detergent fibre (% DM)	32.7	31.6	31.9
Acid detergent fibre (% DM)	19.3	19.1	19.5
PDI (% DM) ^5^	8.6	8.6	8.6
NEF (MJ/kg DM) ^6^	6.64	6.63	6.52
Nutrient composition of YL, WL and RS meal			
Dry matter (% fresh weight)	94.4	93.0	87.6
Crude protein (% DM)	39.5	36.0	36.7
Organic matter (% DM)	93.7	95.2	87.9
Ether extract (% DM)	7.2	13.4	0.4
Neutral detergent fibre (% DM)	35.2	20.8	23.9
Acid detergent fibre (% DM)	19.1	16.5	22.7
PDI (% DM) ^5^	25.2	22.9	23.4
NEF (MJ/kg DM) ^6^	7.53	7.40	6.00
Fatty acid composition of YL, WL and RS meal (g/100 g fatty acids determined)			
C16:0	7.78	8.03	8.56
C18:0	3.81	2.38	1.36
C18:1 n-9	25.4	55.6	45.8
C18:2 n-6	47.5	15.6	29.3
C18:3 n-3	7.90	7.10	6.06

^1^ YL = yellow lupine; ^2^ WL = white lupine; ^3^ RS = rapeseed. ^4^ Contained per 1 kg: CP—867 g, Ca—145 g, P—10 g, Na—60 g, Mg—30 g, S—6 g, Cu—600 mg, Mn—2400 mg, Zn—4000 mg, Se—18 mg, I—60 mg, Co—12 mg, Vitamin A—300,000 IU, Vitamin D3—60,000 IU, Vitamin B1—180 mg, and Vitamin E—720 mg. ^5^ PDI = protein digested in the small intestine [18,19]; ^6^ NEF = net energy of fattening [19].

**Table 2:** Characteristics of the descriptors used in the sensory panel evaluation.

Attribute	Definition	Scale
Beef aroma intensity	The strength of aroma typical for cooked meat	0 = cannot be identified 100 = extremely strong
Off-odour intensity	The strength or richness of unusual odour	0 = cannot be identified 100 = extremely strong
Tenderness	The force required to bite through the sample with molars	0 = very though 100 = very tender
Juiciness	The amount of moisture released by the sample	0 = very low 100 = very high
Fineness	Fineness or coarseness of fibres	0 = very coarse 100 = very fine
Chewability	The amount of residual tissue after most of sample has been masticated	0 = scarcely chewable 100 = easily chewable
Beef flavour intensity	The presence of flavour typical for cooked beef	0 = cannot be identified 100 = extremely strong
Off-flavour intensity	The strength or richness of unusual flavour	0 = cannot be identified 100 = extremely strong
Liver flavour	Flavour typical of cooked liver	0 = cannot be identified 100 = extremely strong
Sour flavour	Strength or richness of sour flavour	0 = cannot be identified 100 = extremely strong
Nutty flavour	Flavour reminiscent of hazelnuts	0 = cannot be identified 100 = extremely strong

**Table 3:** Effects of dietary treatment on growth performance and feed intake.

	Treatment Group				
Trait	YL ^1^	WL ^2^	RS ^3^	SEM	*p*-Value
Slaughter weight (kg)	563.3 ^c^	581.2 ^b^	606.1 ^a^	5.221	<0.001
Daily gain (kg/day)	1.462 ^b^	1.672 ^ab^	1.934 ^a^	0.100	0.014
DM ^4^ intake (kg/day)	10.98	10.75	11.23	0.276	0.118
F:G ^5^ (kg DM/kg gain)	8.16 ^a^	6.49 ^ab^	5.79 ^b^	0.472	<0.001

^1^ YL = yellow lupine; ^2^ WL = white lupine; ^3^ RS = rapeseed; ^4^ DM = dry matter; ^5^ F:G = feed to gain ratio. ^a,b,c^ Values with different superscripts differ at *p* < 0.05.

**Table 4:** Effects of dietary treatment on blood biochemical parameters.

	Treatment Group (Sampling at Day 1)					Treatment Group (Sampling at Day 63)				
Trait	YL ^1^	WL ^2^	RS ^3^	SEM	*p*-Value	YL	WL	RS	SEM	*p*-Value
Total protein (g/L)	65.46	62.79	62.05	1.385	0.177	65.80	65.49	66.05	1.068	0.872
Albumin (g/L)	31.79	32.61	31.68	0.657	0.545	33.09	34.33	34.00	0.383	0.066
Globulin (g/L)	33.67	30.18	30.37	1.356	0.122	32.71	31.16	31.05	1.166	0.508
AGR ^4^	0.97	1.09	1.06	0.051	0.206	1.03	1.11	1.11	0.046	0.303
Creatinine (μmol/L)	119.0	119.0	119.4	4.357	0.997	132.6	136.2	140.7	5.570	0.665
Urea (mmol/L)	3.70	3.81	3.88	0.189	0.772	3.83 ^b^	4.76 ^a^	4.24 ^ab^	0.359	0.043
ALP ^5^ (μkat/L)	2.23	2.67	2.03	0.213	0.105	2.55	2.64	2.34	0.259	0.690
AST ^6^ (μkat/L)	1.59	1.41	1.44	0.213	0.862	1.38	1.26	1.17	0.060	0.057
GGT ^7^ (μkat/L)	0.30	0.30	0.22	0.028	0.073	0.34	0.35	0.34	0.021	0.946
Cholesterol (mmol/L)	1.74	1.76	1.75	0.138	0.996	1.80	1.96	1.91	0.198	0.644
TG ^8^ (mmol/L)	0.16	0.18	0.14	0.012	0.069	0.14	0.16	0.14	0.016	0.694

^1^ YL = yellow lupine; ^2^ WL = white lupine; ^3^ RS = rapeseed; ^4^ AGR = albumin globulin ratio; ^5^ ALP = alkaline phosphatase; ^6^ AST = aspartate aminotransferase; ^7^ GGT = gamma-glutamyl transferase; ^8^ TG = triacylglycerol. ^a,b^ Values with different superscripts differ at *p* < 0.05.

**Table 5:** Effects of dietary treatment on slaughter and carcass traits.

	Treatment Group				
Trait	YL ^1^	WL ^2^	RS ^3^	SEM	*p*-Value
HCW ^4^ (kg)	307.7 ^c^	322.5 ^b^	337.3 ^a^	3.822	<0.001
Dressing percentage	54.77	55.50	55.53	0.382	0.268
Conformation	7.70 ^b^	7.89 ^ab^	8.30 ^a^	0.148	0.024
Fatness	5.10	4.78	5.30	0.167	0.093
Internal fat (kg)	15.89	18.98	17.83	1.443	0.305
Internal fat (% of SW ^5^)	2.83	3.27	2.93	0.247	0.410
LT ^6^ area/100 kg HCW (cm^2^)	12.41	12.53	12.83	0.707	0.901
LT meat (%)	37.81	36.87	36.90	0.865	0.671
Loin fat (%)	3.57	4.14	4.14	0.47	0.592
Loin bones + tendons (%)	27.36	27.33	27.63	0.571	0.915

^1^ YL = yellow lupine; ^2^ WL = white lupine; ^3^ RS = rapeseed; ^4^ HCW = hot carcass weight; ^5^ SW = slaughter weight; ^6^ LT = *longissimus thoracis* muscle. ^a,b,c^ Values with different superscripts differ at *p* < 0.05.

**Table 6:** Effects of dietary treatment on physical meat quality parameters and chemical composition (g/kg muscle) of the *longissimus thoracis* (LT) and *rectus abdominis* (RA) muscles.

	Treatment Group				
Trait	YL ^1^	WL ^2^	RS ^3^	SEM	*p*-Value
LT					
pH	5.59	5.51	5.61	0.034	0.049
Colour L*	40.45	39.88	40.28	0.765	0.799
Colour a*	13.74	13.25	13.35	0.594	0.732
Colour b*	13.47	12.94	13.12	0.457	0.400
Shear force (N/cm^2^)	78.94	73.54	74.57	3.644	0.441
Cooking loss (%)	29.96	29.19	27.80	1.613	0.473
Dry matter (g)	248.2	252.8	245.8	2.02	0.058
Protein (g)	207.3	208.9	208.0	1.09	0.562
Intramuscular fat (g)	21.6	24.9	18.8	2.29	0.171
Total collagen (g)	3.69	3.54	3.68	0.14	0.708
Soluble collagen (%)	26.28	27.22	29.28	2.62	0.220
RA					
pH	5.63	5.59	5.71	0.067	0.314
Colour L*	39.91	41.01	40.65	0.833	0.620
Colour a*	13.23	14.55	14.87	0.714	0.057
Colour b*	13.76	14.64	15.17	0.545	0.099
Shear force (N/cm^2^)	80.8	78.9	83.5	3.53	0.586
Cooking loss (%)	28.53	29.16	31.44	1.488	0.104
Dry matter (g)	241.8	243.6	239.6	3.22	0.299
Protein (g)	203.5	203.1	202.0	1.74	0.506
Intramuscular fat (g)	17.6	20.0	17.7	2.93	0.558
Total collagen (g)	4.7	4.9	5.0	0.28	0.575
Soluble collagen (%)	21.67	20.35	21.53	1.29	0.572

^1^ YL = yellow lupine; ^2^ WL = white lupine; ^3^ RS = rapeseed.

**Table 7:** Effects of dietary treatment on fatty acid proportions (g/100 g fatty acids determined) and contents (mg/100 g muscle) in the *longissimus thoracis et lumborum* muscle.

	Treatment Group					Treatment Group				
Trait	YL ^1^	WL ^2^	RS ^3^	SEM	*p*-Value	YL	WL	RS	SEM	*p*-Value
	Fatty acid proportions					Fatty acid contents				
C14:0	2.75 ^a^	2.70 ^a^	2.49 ^b^	0.057	0.003	33.8 ^ab^	39.2 ^a^	22.9 ^b^	3.706	0.011
C16:0	27.10	27.31	27.41	0.136	0.181	325.9 ^ab^	395.6 ^a^	250.3 ^b^	32.33	0.012
C18:0	17.97 ^a^	18.15 ^a^	16.69 ^b^	0.110	<0.001	216.1 ^ab^	264.0 ^a^	152.6 ^b^	21.92	0.004
C14:1 n-5	0.34 ^b^	0.41 ^a^	0.28 ^c^	0.006	<0.001	4.12 ^ab^	5.91 ^a^	2.56 ^b^	0.478	<0.001
C16:1 n-7	3.18 ^a^	2.69 ^b^	3.08 ^a^	0.036	<0.001	38.4	39.3	28.2	3.805	0.075
C18:1 n-7	1.40 ^b^	1.35 ^b^	1.49 ^a^	0.026	0.003	16.9	19.7	13.7	1.796	0.072
C18:1 n-9	36.66 ^a^	35.95 ^b^	32.85 ^c^	0.1358	<0.001	440.3 ^ab^	520.9 ^a^	300.3 ^b^	42.47	0.003
C18:2 n-6	4.63 ^b^	4.66 ^b^	7.29 ^a^	0.058	<0.001	55.85	67.4	66.7	5.999	0.293
*c*-9, *t*-11 CLA ^4^	0.25 ^a^	0.21 ^b^	0.20 ^b^	0.006	<0.001	3.01 ^a^	3.06 ^a^	1.86 ^b^	0.331	0.019
C18:3 n-3	0.55 ^b^	0.54 ^b^	0.81 ^a^	0.008	<0.001	6.58	7.89	7.48	0.756	0.442
∑SFA ^5^	49.38 ^b^	50.14 ^a^	48.44 ^c^	0.130	<0.001	594.9 ^ab^	727.6 ^a^	442.8 ^b^	60.24	0.008
∑MUFA ^6^	43.12 ^a^	41.95 ^b^	39.23 ^c^	0.119	<0.001	518.4 ^ab^	608.2 ^a^	358.7 ^b^	50.37	0.005
∑PUFA ^7^	7.50 ^c^	7.92 ^b^	12.33 ^a^	0.076	<0.001	90.5	114.9	113.2	10.41	0.175
∑n-6 PUFA	6.54 ^c^	6.86 ^b^	10.66 ^a^	0.070	<0.001	78.9	99.5	97.8	8.984	0.193
∑n-3 PUFA	0.96 ^c^	1.05 ^b^	1.68 ^a^	0.013	<0.001	11.6	15.4	15.4	1.430	0.090
∑FA ^8^						1204 ^ab^	1451 ^a^	915 ^b^	120.7	0.012
∑PUFA/∑SFA	0.15 ^b^	0.16 ^b^	0.26 ^a^	0.002	<0.001					
∑MUFA/∑SFA	0.87 ^a^	0.84 ^b^	0.81 ^c^	0.004	<0.001					
n-6/n-3 ^9^	6.85 ^a^	6.51 ^b^	6.35 ^b^	0.074	<0.001					
IA ^10^	0.75 ^a^	0.77 ^a^	0.73 ^b^	0.009	<0.001					
IT ^11^	1.71 ^a^	1.74 ^a^	1.55 ^b^	0.005	<0.001					

^1^ YL = yellow lupine; ^2^ WL = white lupine; ^3^ RS = rapeseed; ^4^ *c*–9, *t*–11 CLA = *cis*-9, *trans*-11 conjugated linoleic acid; ^5^ SFA = saturated fatty acid; ^6^ MUFA = monounsaturated fatty acid; ^7^ PUFA = polyunsaturated fatty acid; ^8^ FA = total fatty acid; ^9^ n-6/n-3 = ∑n-6 PUFA/∑n-3 PUFA; ^10^ Index of atherogenicity = [C12:0 + (4 × C14:0) + C16:0]/(∑MUFA + ∑PUFA); ^11^ index of thrombogenicity = (C14:0 + C16:0 + C18:0)/[(0.5 × ∑MUFA) + (0.5 × ∑n-6 PUFA) + (3 × ∑n-3 PUFA) + (∑n-3 PUFA/∑n-6 PUFA)]. ^a,b,c^ Values with different superscripts differ at *p* < 0.05.

**Table 8:** Effects of dietary treatment on sensory properties of grilled *longissimus thoracis* (LT) and *rectus abdominis* (RA) muscles.

	Treatment Group				
Trait	YL ^1^	WL ^2^	RS ^3^	SEM	*p*-Value
LT					
Beef aroma intensity	54.2	55.9	51.9	3.83	0.326
Abnormal odour intensity	24.2	22.2	23.9	5.88	0.602
Tenderness	66.1	63.2	59.4	3.23	0.072
Juiciness	59.7 ^ab^	60.3 ^a^	53.1 ^b^	3.39	0.029
Fineness	61.9	63.7	58.8	3.76	0.179
Chewability	62.4	64.8	62.0	3.09	0.573
Beef flavour intensity	56.7	59.9	59.6	3.50	0.277
Abnormal flavour intensity	19.6	20.2	19.9	5.25	0.961
Liver flavour	32.8	35.5	34.7	5.26	0.603
Sour flavour	28.2	29.7	30.2	4.10	0.704
Nutty flavour	60.8	61.4	57.5	3.37	0.289
RA					
Beef aroma intensity	58.1	54.1	58.3	3.70	0.140
Abnormal odour intensity	22.8	21.8	20.4	6.43	0.369
Tenderness	61.9 ^a^	52.3 ^b^	57.4 ^ab^	4.56	0.003
Juiciness	68.4	65.0	63.9	3.78	0.152
Fineness	60.3	55.3	58.2	2.32	0.193
Chewability	59.5 ^a^	53.6 ^ab^	52.2 ^b^	2.65	0.016
Beef flavour intensity	60.7	58.6	59.9	3.89	0.629
Abnormal flavour intensity	21.1	25.0	22.3	6.08	0.122
Liver flavour	32.7	32.7	33.1	5.55	0.978
Sour flavour	25.6	26.4	24.7	4.49	0.800
Nutty flavour	60.6 ^a^	52.4 ^b^	56.0 ^ab^	3.42	0.015

^1^ YL = yellow lupine; ^2^ WL = white lupine; ^3^ RS = rapeseed. ^a,b^ Values with different superscripts differ at *p* < 0.05.

## Data Availability

According to a general policy of the Institute of Animal Science, the data are available upon request. Please contact the corresponding author if you are interested.
